# Analysis of the quadruple *lsu* mutant reveals molecular determinants of the role of LSU proteins in sulfur assimilation in Arabidopsis

**DOI:** 10.1111/tpj.17155

**Published:** 2024-11-29

**Authors:** Justyna Piotrowska, Anna Wawrzyńska, Marcin Olszak, Michal Krzyszton, Anastasia Apodiakou, Saleh Alseekh, José María López Ramos, Rainer Hoefgen, Stanislav Kopriva, Agnieszka Sirko

**Affiliations:** ^1^ Laboratory of Plant Protein Homeostasis Institute of Biochemistry and Biophysics Polish Academy of Sciences Warsaw Poland; ^2^ Laboratory of Seeds Molecular Biology Institute of Biochemistry and Biophysics Polish Academy of Sciences Warsaw Poland; ^3^ Department of Molecular Physiology Max Planck Institute of Molecular Plant Physiology Potsdam Germany; ^4^ Institute for Plant Sciences, Cluster of Excellence on Plant Sciences (CEPLAS) University of Cologne Cologne Germany

**Keywords:** *Arabidopsis thaliana*, sulfur metabolism, LSU (RESPONSE TO LOW SULFUR), sulfite reductase, sulfate deficiency

## Abstract

Because plants are immobile, they have developed intricate mechanisms to sense and absorb nutrients, adjusting their growth and development accordingly. Sulfur is an essential macroelement, but our understanding of its metabolism and homeostasis is limited. LSU (RESPONSE TO LOW SULFUR) proteins are plant‐specific proteins with unknown molecular functions and were first identified during transcriptomic studies on sulfur deficiency in Arabidopsis. These proteins are crucial hubs that integrate environmental signals and are involved in the response to various stressors. Herein, we report the direct involvement of LSU proteins in primary sulfur metabolism. Our findings revealed that the quadruple *lsu* mutant, q‐lsu‐KO, which was grown under nonlimiting sulfate conditions, exhibited a molecular response resembling that of sulfur‐deficient wild‐type plants. This led us to explore the interactions of LSU proteins with sulfate reduction pathway enzymes. We found that all LSU proteins interact with ATPS1 and ATPS3 isoforms of ATP sulfurylase, all three isoforms of adenosine 5´ phosphosulfate reductase (APR), and sulfite reductase (SiR). Additionally, *in vitro* assays revealed that LSU1 enhances the enzymatic activity of SiR. These results highlight the supportive role of LSU proteins in the sulfate reduction pathway.

## Significance Statement

This study demonstrates that the quadruple lsu mutant exhibits impaired sulfate assimilation and reduction at the transcript, metabolite, and long‐distance sulfur flux levels. Our subsequent research has revealed the molecular mechanism underlying these findings, showing a direct interaction between LSU proteins and enzymes involved in sulfate reduction, particularly sulfite reductase (SiR), with LSU1 enhancing SiR activity.

## INTRODUCTION

Sulfur (S) is an essential macroelement for plant life and has numerous biological functions (Leustek et al., 2000). The main source of S in the environment is inorganic sulfate. Sulfate is absorbed by plants through sulfate transporters (SULTRs) and further assimilates into organic S compounds (Takahashi, 2010; Zhang et al., 2020). Sulfate assimilation takes place in the plastids, mainly in the leaves, and begins with the activation of ${\mathrm{SO}}_{4}^{2-}$ to adenosine 5′‐phosphosulfate (APS) by ATP sulfurylase (ATPS). APS is reduced to sulfite by APS reductase (APR) and further to sulfide by sulfite reductase (SiR). Cysteine is synthesized by *O*‐acetylserine (thiol) lyase from sulfide and *O*‐acetylserine (OAS), which is formed in a reaction between serine and acetyl‐CoA catalyzed by serine acetyltransferase (SAT). Cysteine is the first compound containing reduced S in the S assimilation pathway and serves as a precursor for other organic S compounds, such as methionine and glutathione (GSH) (Romero et al., 2014). GSH is the most abundant metabolic thiol component in the cell and is widely used in plant stress defense, redox regulation, S storage, and transport (Li et al., 2020). Other S‐containing compounds include glucosinolates (GSL), which are major secondary metabolites synthesized by the Brassicaceae family, including *Arabidopsis thaliana*. GSL are the primary defense compounds against herbivores and pathogens and act as S‐storage sources (Aarabi et al., 2016; Burow & Halkier, 2017).

In plants subjected to S limitation, the amount of internal sulfate decreases, followed by decreases in cysteine, GSH, and GSL levels (Hirai et al., 2003; Nikiforova et al., 2003). Inhibition of cysteine biosynthesis also leads to the accumulation of its direct precursor OAS (Hirai et al., 2003; Nikiforova et al., 2003, 2005). In response to S‐deficient conditions, plants stimulate sulfate uptake and assimilation and the degradation of GSH and GSL with simultaneous repression of GSL biosynthesis. During the catabolism of GSL, sulfate is released and available for reincorporation into essential S‐containing compounds of primary metabolism.

The response to S deficiency is primarily controlled through transcriptional regulation (Maruyama‐Nakashita et al., 2006). An integrative meta‐analysis of transcriptomic data from five different experiments available in public databases revealed a robust set of genes whose expression depends on sulfate availability (Henriquez‐Valencia et al., 2018; Maruyama‐Nakashita et al., 2005). The biological functions of approximately 45% of the proteins encoded by these genes are unknown. Among them are three genes from the *LSU* (*RESPONSE TO LOW SULFUR*) family, namely, *LSU1*, *LSU2*, and *LSU3*.

LSUs are plant‐specific proteins of unknown function that were initially identified during transcriptomic studies of the S deficiency response in Arabidopsis (Maruyama‐Nakashita et al., 2005). The genome of *A. thaliana* encodes four LSU proteins (LSU1, AT3G49580; LSU2, AT5G24660; LSU3, AT3G49570; and LSU4, AT5G24655). A recent study revealed that the mRNA levels of all *LSU* genes are increased under S deficiency in roots and leaves (Uribe et al., 2022). The *LSU* genes and their homologs are upregulated by S deficiency not only in Arabidopsis but also in tobacco (Lewandowska et al., 2010), tomato, and wheat (Uribe et al., 2022). Additionally, *LSU1* was identified as a part of the “OAS cluster,” six genes that are induced when OAS accumulates (Hubberten et al., 2012). Recently, an extended OAS cluster coexpression network was proposed, which includes *LSU3* (Apodiakou & Hoefgen, 2023). Moreover, LSU proteins have been associated with the metabolism of GSL and their possible function in adjusting S flow from GSL to sulfate during S deficiency and cadmium stress (Li et al., 2023; Yang et al., 2023).

In a recent study, we described the generation and phenotyping of a set of *lsu* knockout mutants, including a quadruple *lsu* mutant (q‐lsu‐KO) (Piotrowska et al., 2024). This study revealed that LSU proteins are not essential for plant growth or reproduction but instead act as modulators of the S deficiency response. Here, we analyzed the q‐lsu‐KO mutant at the molecular level to determine the role of LSU proteins in S metabolism via a global transcriptomic and metabolomic approach as well as S assimilation flux analysis. Moreover, we tested the interactions of LSU proteins with enzymes of the sulfate assimilation pathway and investigated the impact of LSU1 on SiR activity *in vitro*, which revealed that LSU proteins act through protein–protein interactions.

## RESULTS

### Transcript profiling of q‐lsu‐KO seedlings reveals differences in the expression of S metabolism genes

To dissect the role of LSU proteins in plant metabolism, especially S‐metabolism, we investigated the impact of knocking out all four *LSU* genes on the plant global transcriptome under both normal [nS] and S deficiency [dS] conditions via RNA sequencing (3′RNA‐seq). Libraries were generated from total RNA extracted from either the shoots or roots of 15‐day‐old q‐lsu‐KO mutant and wild‐type (WT) plants. The plants were grown under [nS] conditions for 10 days and then transferred to either [nS] or [dS] conditions for another 5 days. We decided to apply short‐term “induced S starvation” to avoid secondary changes in transcriptome (Nikiforova et al., 2003).

**Figure 1:**
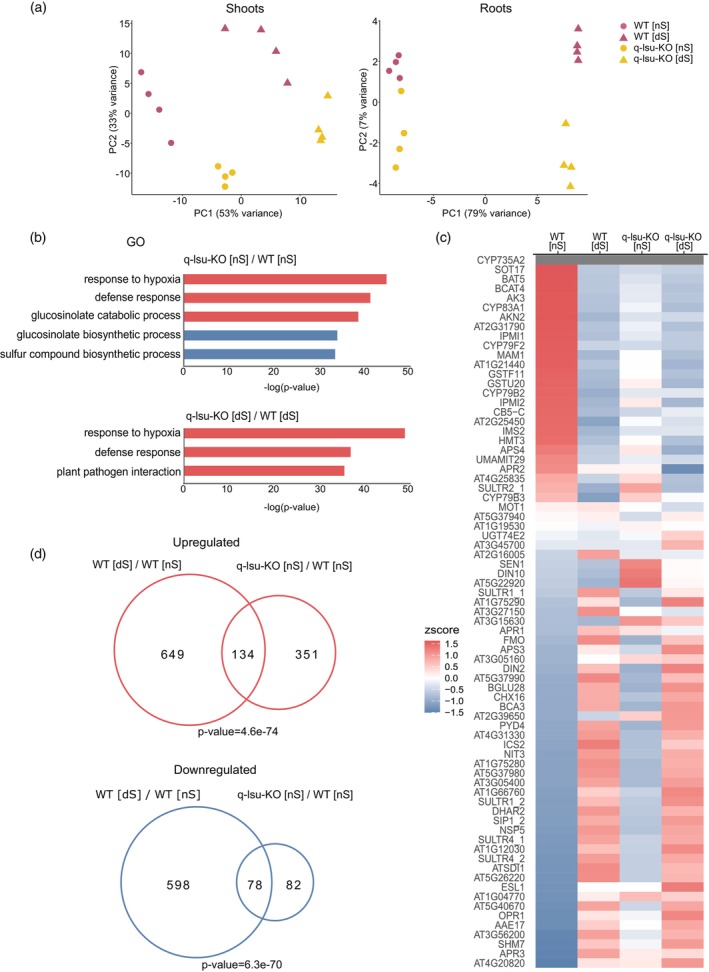
Analyses of DEGs in WT and q‐lsu‐KO mutant shoots and roots grown under normal conditions [nS] and 5‐day S deficiency [dS] conditions. (a) PCA plot of the transcriptome profiles of WT and q‐lsu‐KO mutant shoots and roots; each dot represents one biological replicate. (b) Selected GO‐term enrichment analysis results of the shoot DEGs for the q‐lsu‐KO mutant grown in [nS] (upper) and [dS] (lower) conditions. (c) Heatmap showing the regulation of S deficiency marker genes (the mean z score). (d) Venn diagrams showing the overlap of genes with upregulated or downregulated expression either in response to 5 days of S deficiency in the WT or changed in the q‐lsu‐KO mutant in [nS] conditions. The number of DEGs (1.5‐fold difference at *P* ≥ 0.05) is shown for each set. Significance of overlap was calculated using GeneOverlap R package with the background set to genes detected in the experiment.

Principal component analysis (PCA) revealed the formation of coherent clusters among biological replicates both for root and shoot samples (Figure 1a). This indicates the high reproducibility of the experiment and significant differences between biological samples. The first principal component (PC1) separated samples with different S levels, whereas the PC2 was rather related to the genotype.

Differentially expressed genes (DEGs) between genotypes and between conditions were examined via DESeq2 (*P*adj>0.05; FC >1.5). The list of DEGs is shown in Table S1. S deficiency leads to profound changes in gene expression in Arabidopsis. 3′RNA‐seq analysis revealed 783 and 870 genes with upregulated and 676 and 622 genes with downregulated expression under [dS] conditions in the shoots of the WT and q‐lsu‐KO mutant, respectively (Figure S1a). In the roots, the number of DEGs was lower; 401 and 323 genes had an upregulated and 462 and 471 had a downregulated expression in the WT and q‐lsu‐KO mutant, respectively (Figure S1b). As expected, among the genes with an upregulated expression in both genotypes were S deficiency marker genes such as *SULFUR DEFICIENCY INDUCED1* (*SDI1*), *BETA‐GLUCOSIDASE 28* (*BGLU28*), and *GAMMA‐GLUTAMYL CYCLOTRANSFERASE2;1* (*GGCT2;1*) (Henriquez‐Valencia et al., 2018). These findings indicate that both WT and q‐lsu‐KO plants respond to S deprivation by modulating their transcriptomes.

Compared with WT, the deletion of all LSU genes led to significant upregulation of 485 and 497 genes' expression and downregulation of 160 and 47 genes' expression in shoots under [nS] and [dS] conditions, respectively. In the roots, there were fewer changes, and there were only 17 genes with upregulated and 9 genes with downregulated expression in [nS] and 13 with upregulated and 6 with downregulated expression in [dS] conditions (Figure S1a,b).

To determine the common functions of these DEGs, GO (Gene Ontology) analysis was performed (Figure 1b). We noticed that in shoots under [nS] conditions, a lack of LSU proteins caused the increased expression of genes associated with responses to hypoxia, the defense response, and GSL catabolic processes. Interestingly, the genes corresponding to the latter phenomena were similarly regulated during [dS] in the WT. Under [dS] conditions, genes associated with responses to hypoxia and the defense response but not genes involved in the GSL metabolic process were also upregulated. The genes downregulated under [nS] conditions were enriched in the biosynthesis of S compounds, particularly GSL. However, no GO category was enriched in root tissue under either condition because of the small number of regulated genes. The detailed list of GO categories is provided in Table S2.

Additionally, the changes observed in our transcriptomic data were compared with 75 genes selected as marker genes of S deficiency (Henriquez‐Valencia et al., 2018) (Figure 1c). Interestingly, the expression of many of these genes changed in the q‐lsu‐KO mutant already under [nS] conditions. A comparison of the expression of these selected 75 genes in the q‐lsu‐KO mutant to that in the WT plants revealed that 49 genes were altered in [nS] and 13 in [dS] conditions. The genes with an upregulated expression in the q‐lsu‐KO mutant included genes related to the uptake and assimilation of sulfate, such as the sulfate transporters SULTR2;1, SULTR4;2, and APR3, whereas the genes with a downregulated expression were related to the synthesis of aliphatic GSL, for example, SOT17, BAT5, and CYP79F2. Hence, the gene expression pattern observed in the mutant under [nS] conditions mirrored that of WT plants experiencing S starvation. Therefore, we compared the effects of S starvation on gene expression in WT to the effects of *LSU* gene deletions (Figure 1d). The Venn diagrams shown herein clearly illustrate the significant overlap of genes altered in the q‐lsu‐KO mutant in [nS] conditions with gene expression changes in the WT in [dS] conditions. Namely, in the overlapping sections there are mainly genes connected with S‐metabolism pathways, like sulfate transport and reduction, glutathione metabolism, and glucosinolate synthesis and degradation. This confirms that despite probable secondary effects caused by both S depletion and *lsu* mutations, the transcriptomic differences between these conditions are similar underlying the LSU function in S metabolism.

### Global overview of the metabolomic profile

**Figure 2:**
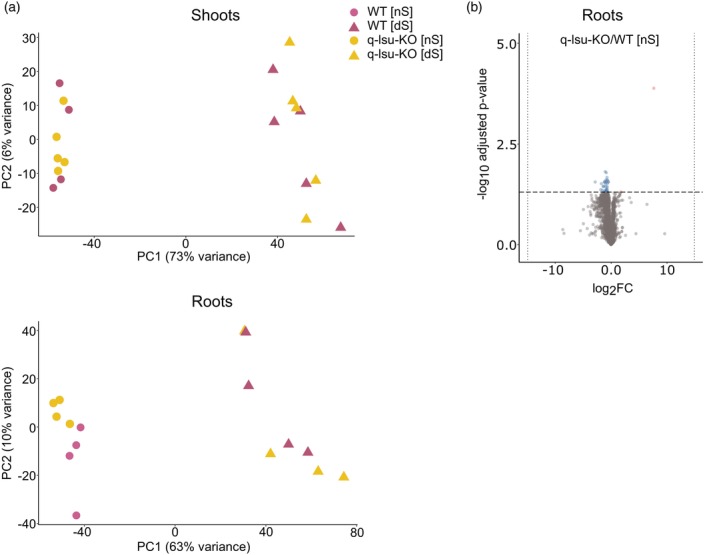
Comparative analysis of metabolome profiles of WT and q‐lsu‐KO mutant shoots and roots under normal conditions [nS] and 5‐day S deficiency [dS] conditions. (a) PCA plot of metabolome profiles of WT and q‐lsu‐KO mutant shoots and roots; each dot represents one biological replicate. (b) Volcano plot showing the comparison of metabolite levels in the roots of the q‐lsu‐KO mutant versus WT under [nS] conditions. The fold change (log2) is plotted on the x‐axis, and the adjusted *P* value (negative log10) is plotted on the y‐axis. The light gray dots represent all the metabolites, while the color dots represent significantly changed metabolites (fold change ≥1.5 and *P* ≤ 0.05): red indicates increased levels, whereas blue indicates decreased levels.

Next, we determined the metabolic changes in the q‐lsu‐KO mutant via global metabolic profiling using LC–MS of the shoots and roots of the q‐lsu‐KO mutant and WT plants grown constantly for 14 days under [nS] and [dS] conditions. In this case, we decided to use more severe S‐starvation conditions (“constitutive S starvation”) than for transcriptomic analyses to induce stronger changes in metabolite levels (Nikiforova et al., 2003, 2005). A total of 8300 metabolic features were detected, and after the exclusion of low‐variance (40%) and low‐abundance (20%) features, analyzed for the leaves and roots separately. The processed data with FDR and log2 fold‐change values are available in Table S3. PCA revealed that sample grouping was dependent on S status and genotype, although for the latter, sample grouping only occurred in the roots under [nS] conditions (Figure 2a). This result is different from that of the PCA of the transcriptomics data (Figure 1a). This finding suggests that metabolome changes are driven primarily by S availability, with a minimal effect of the deletion of *LSU* genes. Significant changes in the levels of metabolites in WT and q‐lsu‐KO mutant plants and both parts of the plants were detected between conditions, as shown by volcano plots (Figure S2a,b). Among all the detected metabolite features, some were assigned to specific compounds, including 10 aliphatic GSL. However, their levels did not differ significantly between the q‐lsu‐KO mutant and WT, despite the observed downregulation of the expression of the genes involved in GSL synthesis in q‐lsu‐KO mutant (Figure 1b,c) Under [nS] conditions, the q‐lsu‐KO mutant presented a significant (FDR <0.05, log2 FC >1.5) reduction in the levels of only 39 metabolites in the roots, whereas 2 metabolites presented increased levels (Figure 2b). However, the identities of these metabolites are yet unknown. Notably, the effects of *LSU* gene deletions manifested at the transcriptome level mainly in the leaves but at the metabolome level in the roots. Under [dS] conditions, no metabolites exhibited significant changes in the shoots or roots of the q‐lsu‐KO mutant compared with those of the WT (Figure 2a,b).

### LSU gene deletions affect the accumulation of S‐containing metabolites

**Figure 3:**
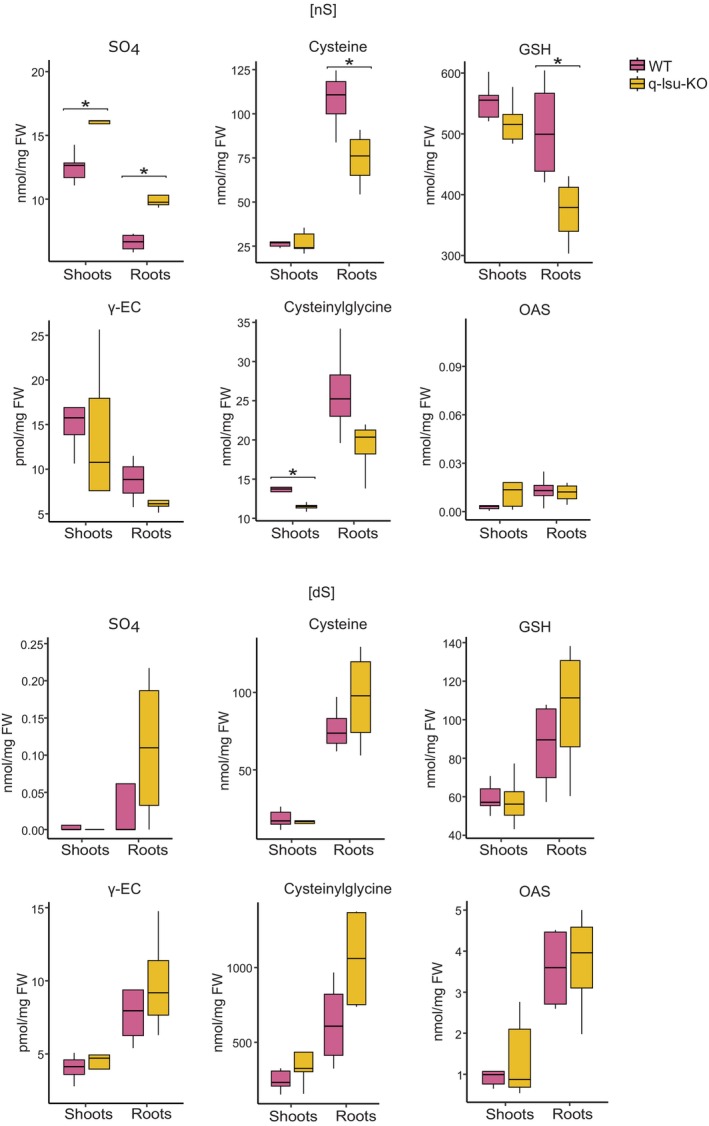
Levels of metabolites in the shoots and roots of WT and q‐lsu‐KO mutant plants grown for 14 days under either S‐sufficient [nS] or S‐deficient [dS] conditions. The data are presented as box plots (25–75%) of four biological replicates, with the inside lines indicating the medians and whiskers indicating 1.5 interquartile range (IQR). Statistical differences between the WT and q‐lsu‐KO mutant plants were analyzed via Student's *t* test (*P* values ≤0.5 were considered significant) and are marked by asterisks. GSH, glutathione; OAS, O‐acetylserine; SO_4_, sulfate; γ‐EC, gamma‐glutamylcysteine.

There were no significant effects of *LSU* gene deletions on the metabolome at the global level; therefore, we investigated the response of S‐related compounds in a more direct approach. S‐related metabolites were quantified in the q‐lsu‐KO mutant and the WT plants after 14 days of growth under either [nS] or [dS] conditions (Figure 3). Under [nS] conditions, the q‐lsu‐KO mutant presented a 30% increase in sulfate accumulation in shoots and a 50% increase in roots compared with those of the WT. Despite the increased sulfate levels, the average levels of organic S compounds, including cysteine, GSH, the GSH precursor gamma‐glutamylcysteine (γ‐EC), and the catabolic by‐product of GSH cysteinylglycine, were reduced (although not significantly different for the latter two) in the roots of the q‐lsu‐KO mutant under the same conditions (Figure 3). The deletion of *LSU* genes did not affect the OAS level, implying that the reduced levels of cysteine and GSH were not due to OAS deficiency. Under [dS] conditions, significant differences in metabolite levels between the genotypes were not observed.

### Uptake and assimilation of sulfate are increased in the q‐lsu‐KO mutant

**Figure 4:**
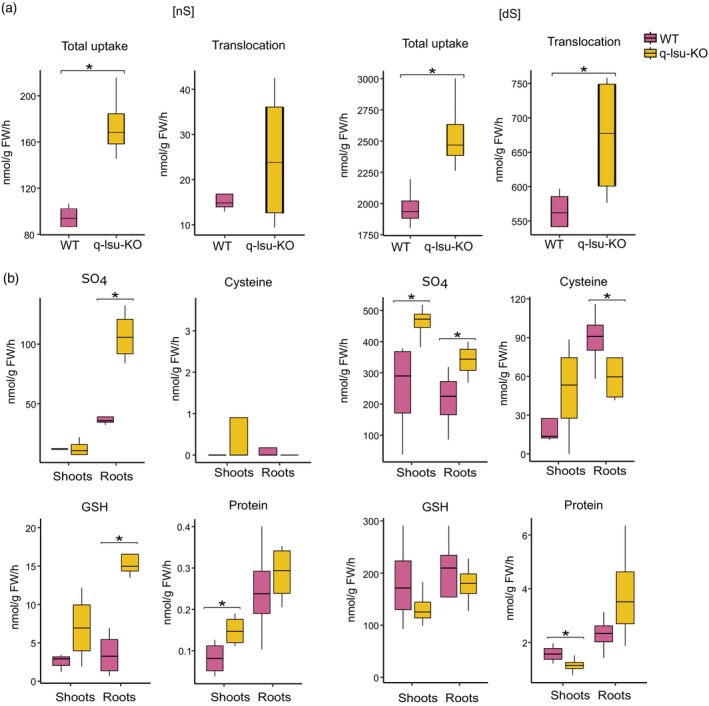
[^35^S] sulfate uptake and partitioning in WT and q‐lsu‐KO mutant plants. The plants were grown for 14 days on either 0.75 mM MgSO_4_ under [nS] conditions or 0.015 mM MgSO_4_ under [dS] conditions and subsequently fed [^35^S] sulfate. The data are presented as box plots (25–75%) of four biological replicates, with the line inside marking the median and whiskers indicating 1.5 interquartile range (IQR). Statistical differences between the WT and q‐lsu‐KO mutant plants were analyzed via Student's *t* test (*P* values ≤0.5 were considered significant) and are marked by asterisks. (a) Sulfate uptake and the root‐to‐shoot translocation level of sulfate with ^35^S. (b) The level of metabolites labeled with ^35^S.

The increased sulfate levels in the roots and shoots of the q‐lsu‐KO mutant under [nS] conditions indicate the increased capacity of its uptake; therefore, sulfate uptake and assimilation rates were assessed in the q‐lsu‐KO mutant and WT plants cultivated either under conditions of normal sulfate or limiting sulfate for 14 days and subsequently fed [^35^S] sulfate for 4 h. The incorporation of ^35^S into key S‐containing compounds, such as cysteine, GSH, and protein, along with overall sulfate uptake and its translocation from the roots to the shoots, was examined. During S deficiency, Arabidopsis plants typically exhibit an increase in the sulfate uptake capacity of their roots and increased sulfate translocation to their shoots *via* sulfate transporters. In the q‐lsu‐KO mutant, there was a significant increase in sulfate uptake, with levels rising by more than 80% under [nS] conditions and nearly 30% under [dS] conditions compared with those in the WT (Figure 4). Increased sulfate uptake directly resulted in its translocation to the leaves, resulting in increases observed under [nS] and [dS] conditions, but these increases were statistically significant only under the [dS] condition. Consequently, the levels of radioactive sulfate were greater in the shoots and roots of the q‐lsu‐KO mutant than in those of the control plants. Despite the substantial increase in sulfate uptake in the q‐lsu‐KO mutant, the incorporation of ^35^S into cysteine, GSH, and protein was not significantly elevated and even decreased (Figure 4). Only slightly increased flux into proteins was observed in the shoots, whereas GSH was observed in the roots of the q‐lsu‐KO mutant grown under [nS] conditions. In contrast, a decreased flux of ^35^S into the protein in shoots and cysteine in roots was observed in plants grown in [dS] conditions.

### LSU proteins interact with enzymes of the assimilatory sulfate reduction pathway

**Figure 5:**
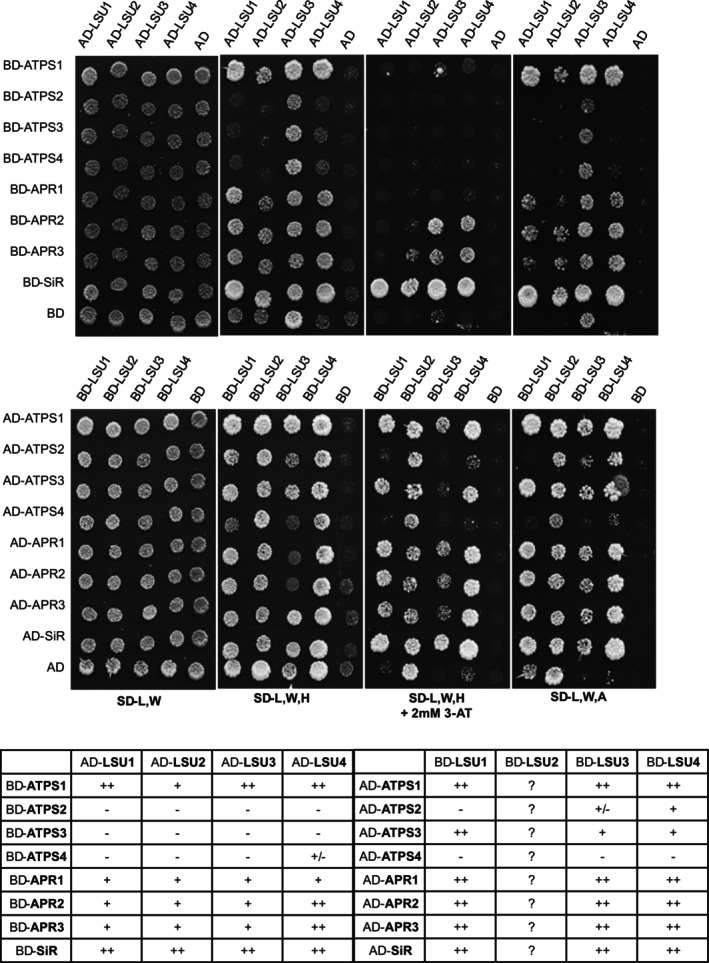
Interactions between LSUs and the enzymes of the sulfate reduction pathway in Y2H experiments. A summary of interactions is shown below the images, illustrating the growth of yeast cultures on different selection media. The plates were incubated for 3 days at 30°C. “++,” “+,” or “+/−” reflects the strength of the interaction; “++” refers to growth on all the selective plates, whereas “+/−” means that growth was observed only under very weak selection pressure. The autoactivation results for BD‐LSU2 and LSU3 are marked by question marks (?).

**Figure 6:**
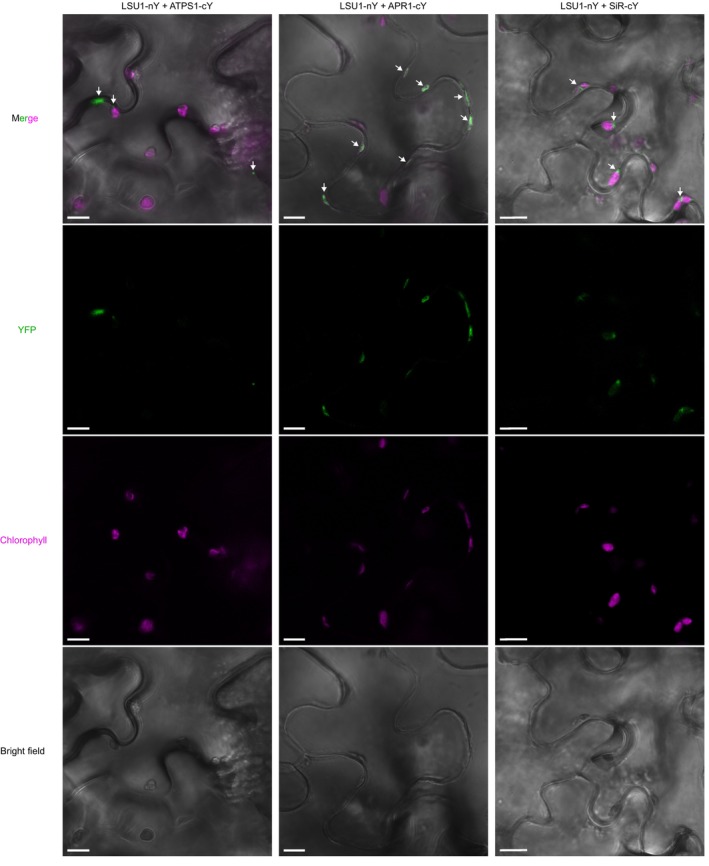
A representative biomolecular fluorescence complementation (BiFC; green spots, marked by arrows) image of LSU1 with APS1, APR1, and SiR. cY and nY represent the C‐terminal and N‐terminal sections of YFP, respectively, fused to the indicated protein. Scale bar: 10 μm.

The transcriptomics data together with the S‐related metabolite data (sulfate accumulation in contrast to a lower level of cysteine) for the q‐lsu‐KO mutant grown in [nS] conditions suggested a lower efficiency of sulfate reduction to sulfide. Previous biomolecular fluorescence complementation (BiFC) experiments have shown that ATPS1 can interact with all four LSU proteins in *Nicotiana benthamiana* leaves (Niemiro et al., 2020), suggesting the possibility of an additional level of regulation of sulfate reduction. Indeed, LSU1 colocalizes with chloroplasts, where it interacts with the superoxide dismutase FSD2 (Garcia‐Molina et al., 2017) and where all the enzymes of the sulfate reduction pathway act (Schwenn & Depka, 1977). To determine whether the LSU proteins can interact with other enzymes of the pathway, we used a Y2H screen. The interactions were tested in two different sets: in the first set, the GAL4 BD domain was fused to eight enzyme isoforms (four ATPS, three APR, and SiR), while the LSU proteins were fused with the GAL4 AD domain, and in the second set, the GAL4 domains were switched between protein pairs (Figure 5). The results of the screen revealed that all the enzymes, except ATPS2 and ATPS4, bind to all four LSU proteins. The strongest interactions were observed between LSU proteins and ATPS1 and SiR. To verify the interactions via other methods, we performed *in planta* BiFC assays with LSU1 and ATPS1, APR1, and SiR (Figure 6). The BiFC assay confirmed the interaction between selected proteins. In some cases, we observed the fluorescence of the interacting proteins in close proximity or inside chloroplasts. However, most of the interactions were localized to the cytosol. The negative controls, that is, the C‐terminal or N‐terminal sections of YFP coexpressed with appropriate protein fusions, are shown in Figure S3.

### LSU1 increases the activity of SiR

**Figure 7:**
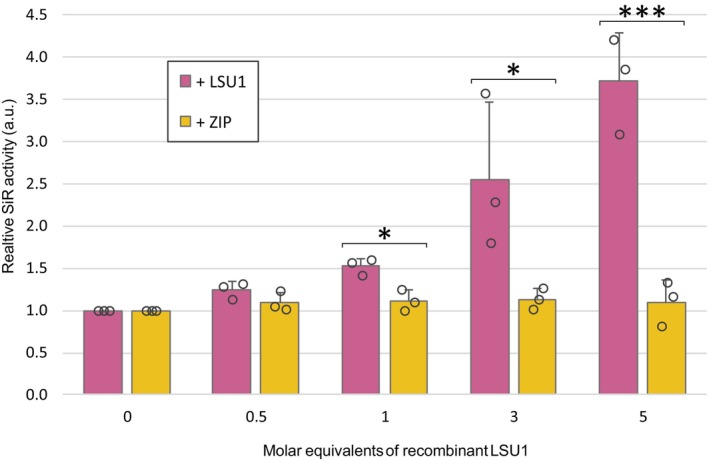
Relative *in vitro* activity of recombinant His–SiR in the presence of increasing concentrations of His–LSU1. The error bars correspond to the SDs of three independent experiments, and significant differences from the controls were assessed via Student's *t* test (*P ≤ 0.05; ***P ≤ 0.001). The auxiliary unit (a.u.) is the number of nmoles of cysteine produced during 60 min of the assay.

Considering that the absence of LSU proteins results in the accumulation of sulfate and reduced cysteine production under [nS] conditions, we wondered whether LSU proteins might play a direct role in stimulating the activities of enzymes in the sulfate reduction pathway. Thus, we examined the interaction between SiR and LSU1. SiR has a single isoform and shows the strongest interaction with LSU proteins based on Y2H results (Figure 5). The *sir1‐1* mutant showed characteristics similar to those of the q‐lsu‐KO mutant concerning the expression of S metabolism genes, as well as increased sulfate levels in roots and shoots and reduced cysteine levels in roots (Dong et al., 2017). For the measurement of SiR activity, we decided to use an *in vitro* assay with recombinant His‐tagged SiR and LSU1 produced and purified from *Escherichia coli* (Figure S4). The effect of LSU1 on SiR activity was examined via the use of the noninteracting ZIP protein to assess specificity. The ZIP protein is a DNA‐binding domain of ABF3 that is similar in size to LSU1 but is not a partner of SiR (Figure S5). The functionality of His‐tagged SiR was confirmed by plotting SiR activity against the enzyme concentration, which yielded a typical saturation curve (Figure S6). Nonsaturating enzyme concentrations were then incubated with increasing amounts of His–LSU1, and SiR activity was determined (Figure 7). Recombinant LSU1 specifically induced SiR enzyme activity, whereas the addition of noninteracting ZIP did not affect SiR activity (Figure 7). The addition of LSU1 to the reaction gradually increased SiR activity during the incubation time (Figure S7). Therefore, mechanistically, LSU1 can increase sulfide production by directly interacting with and activating SiR.

### LSU1 binds to the iron–sulfur cluster‐binding region of SiR

**Figure 8:**
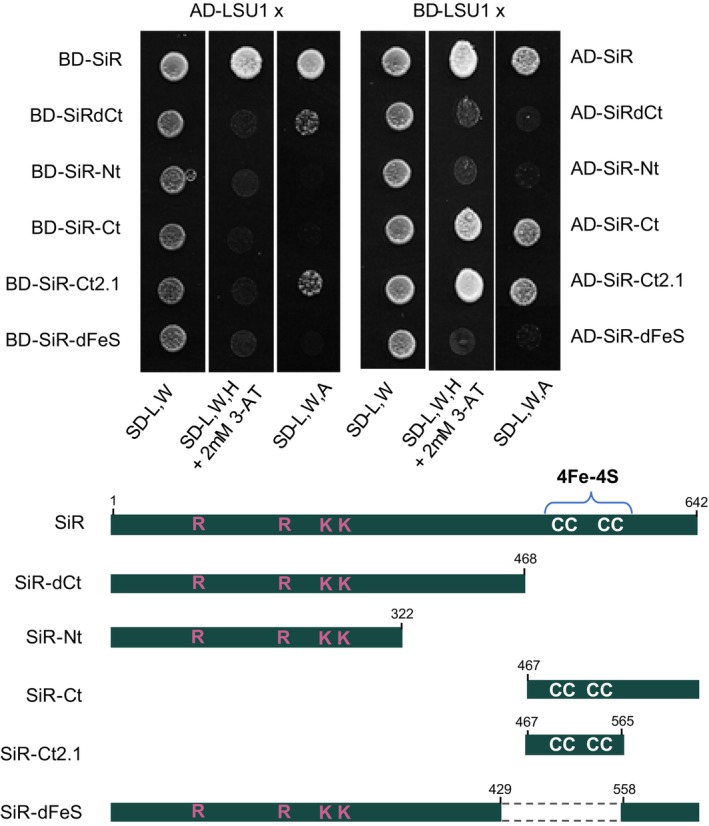
Interactions between LSU1 and different SiR truncations in Y2H experiments. The growth of yeast cultures on different selection media is shown. Illustrated below are the different truncations of SiR used in the experiment. Basic amino acids crucial for binding sulfite are marked in red, while cysteines of the iron–sulfur cluster (4Fe–4S) are marked in white.

To determine which region of SiR is involved in the interaction with relatively small proteins such as LSU proteins, we generated different SiR protein truncations and performed additional Y2H assays. While LSU1 did not interact with the N‐terminal region of SiR (1–322), it strongly bound the C‐terminal domain (467–642) (Figure 8). We further narrowed the interaction region down to the region of amino acids (aa) 467–565 of SiR, which contains four cysteine residues crucial for binding the iron–sulfur cluster [4Fe–4S] (Figure 8). One of the cysteines also serves as an axial ligand to the siroheme (Nakayama et al., 2000). SiR uses a [4Fe–4S] cluster alongside a siroheme to catalyze the six‐electron reduction reaction of sulfite to sulfide. The physiological donor of the six electrons in plants is ferrodoxin (Nakayama et al., 2000). Indeed, the SiR protein with the deletion of the region binding the [4Fe–4S] cluster was not recognized by LSU1.

## DISCUSSION

*LSU* genes have been identified via transcriptomic studies as being induced during S deficiency in Arabidopsis, but their molecular function in response to starvation has remained unclear. In this work, we report the supportive role of LSU proteins in the sulfate reduction pathway. RNA‐seq data revealed an impact of LSU proteins on the plant transcriptome under S starvation and normal S availability conditions. Notably, the DEG profile of the q‐lsu‐KO mutant under normal conditions partially mirrored the response observed in WT plants under S deficiency conditions. GO analysis highlighted alterations in genes associated with the metabolism of S‐containing compounds under sufficient S conditions. Intriguingly, the q‐lsu‐KO mutant presented increased expression of genes related to the plant defense response. This GO term is intricately linked to S metabolism, involving GSH and GSL as the primary line of defense against various biotic and abiotic stressors (Aarabi et al., 2016; Burow & Halkier, 2017; Li et al., 2020). Additionally, transcriptome analysis of the q‐lsu‐KO mutant revealed the upregulation of genes involved in the response to hypoxia. A similar phenomenon was observed in transcriptomic analyses of the slim1 mutant (Dietzen et al., 2020). SLIM1 is a crucial transcription factor of the Arabidopsis response to S deficiency and a direct positive regulator of *LSU* gene expression (Maruyama‐Nakashita et al., 2006; Wawrzynska et al., 2010). Similar to that of the *slim1* mutant, transcriptomic analysis of the q‐lsu‐KO mutant under [nS] conditions revealed altered expression of genes that are markers of S deficiency, for example, *SULTR1;1*, *SULTR2;1, APR3*, and *GGCT2;1*, and genes involved in GSL synthesis and degradation, particularly aliphatic GSL known for their sensitivity to S deprivation (Maruyama‐Nakashita, 2017), such as *SDI1*, *BGLU28*, *MAM1*, *BCAT4*, and *CYP79F1*. One can speculate about the hierarchy of signaling events and whether LSU proteins are upstream or downstream of SLIM1. As the lack of LSU proteins causes similar changes in gene expression driven by SLIM1 in response to [dS] conditions, we could assume that LSU proteins may have some regulatory functions in these transcription events. However, in the promoters of three LSU genes, there are direct binding sites for SLIM1, enabling their strong induction during S deficiency (Maruyama‐Nakashita et al., 2006, Wawrzynska et al., 2010). In contrast to q‐lsu‐KO mutant, in [nS] conditions the *slim1* mutant accumulates nearly nine times more OAS than WT plants, but significantly less sulfate (Wawrzynska et al., 2022). However, under [dS] conditions, the *slim1* mutant accumulated 70 times more sulfate in its shoots compared to the WT plants, a feature not observed in q‐lsu‐KO mutant. SLIM1 is a transcription factor that regulates the expression of numerous genes, not only *LSU*, which explains the distinct metabolic characteristics observed between these mutants. Moreover, a compelling observation was the significant overlap between DEGs in the q‐lsu‐KO mutant compared with the WT plants under [nS] conditions and DEGs in the WT plants grown under [dS] conditions compared with the WT plants under [nS] conditions (Figure 4d). Although the overlapping genes include less obvious examples, such as those encoding proteins involved in leucine biosynthesis, phosphate transport, and response to jasmonic and salicylic acids, the majority of these genes are related to S metabolism. This specific response suggests that the q‐lsu‐KO mutant senses sulfate limitation despite its adequate supply. Our findings are in agreement with previously reported observations, where we showed that the q‐lsu‐KO mutant presented reduced shoot growth and enhanced root elongation under [nS] conditions, which are common symptoms of S deficiency (Kutz et al., 2002). The observed root elongation might be attributed to the increased expression of the *NIT3* gene observed in the q‐lsu‐KO mutant (interestingly, a significant difference was detected only in the shoots) (Table S1). *NIT3* encodes one of four Arabidopsis nitrilases responsible for the metabolism of nitriles originating from GSL breakdown (Vorwerk et al., 2001). NIT3 converts indole‐3‐acetonitrile to the plant growth hormone indole‐3‐acetic acid (Bartling et al., 1994). S deprivation not only triggers *NIT3* expression specifically but also accelerates the breakdown of glucobrassicin, a GSL in roots, which is a precursor of indole‐3‐acetonitrile. This, in turn, elevates the auxin level, promoting root elongation and branching, thus increasing the chances of plants accessing fresh supplies of this essential element (Kutz et al., 2002).

PCA of the metabolomics data revealed that changes in the metabolome are mostly due to the availability of S, whereas deleting the LSU genes does not seem to have much impact. Hence, the clearly visible transcriptome effects of the lack of LSU proteins are probably counteracted by other regulatory mechanisms to keep the metabolome in its appropriate state. With respect to the transcriptomics data, analysis of S‐related metabolite levels also revealed that the q‐lsu‐KO mutant exhibited a response characteristic of S deficiency even in an [nS] environment. Under [dS] conditions, in WT plants, we observed a decrease in the levels of sulfate, cysteine, and GSH, whereas the level of cysteinylglycine, a glutathione breakdown product, increased. However, the q‐lsu‐KO mutant under [nS] conditions presented higher sulfate levels, likely due to increased expression of *SULTR* genes, but presented lower levels of cysteine and GSH than did the WT. This result is consistent with measurements of the uptake, translocation, and assimilation of S using radiolabeled ^35^S. There was increased sulfate uptake in the q‐lsu‐KO mutant, resulting in elevated sulfate levels. Despite the significant increase in sulfate uptake, the incorporation of ^35^S into protein and GSH was only slightly greater than that in the WT plants grown under [nS] conditions. The incorporation of ^35^S under [dS] conditions did not significantly increase in the q‐lsu‐KO mutant and even decreased in cysteine and proteins. These results again suggest that *LSU* deletion impairs sulfate assimilation and reduction. Notably, OAS concentration that grows rapidly in [dS] conditions is unchanged in q‐lsu‐KO mutant compared to WT in [nS] conditions (Figure 3). OAS has been for a long time controversially discussed as a mediator of plant S status (Apodiakou & Hoefgen, 2023; Hirai et al., 2003; Hopkins et al., 2005). OAS treatment leads to enhanced sulfate uptake and reduction rate, driven by elevated transcript levels of the associated genes (Hirai et al., 2003; Neuenschwander et al., 1991). Since these traits are also triggered by S deficiency and OAS accumulates in S‐deficient plants, OAS was initially thought to be involved in S deficit signaling. However, other studies have shown that the increase in transcript levels precedes the increase in OAS level, raising questions about its role in sulfur signaling (Hopkins et al., 2005). In this study, we demonstrate that the transcripts associated with S deficiency are affected by the absence of LSU proteins, even though the OAS level remains unchanged. Interestingly, *LSU1* and *LSU3* belong to the ‘OAS cluster’, a group of genes that are induced in response to OAS accumulation (Apodiakou & Hoefgen, 2023; Hubberten et al., 2012). A schematic summary of the S‐related gene and metabolite changes described in this work is shown in Figure S8.

**Figure 9:**
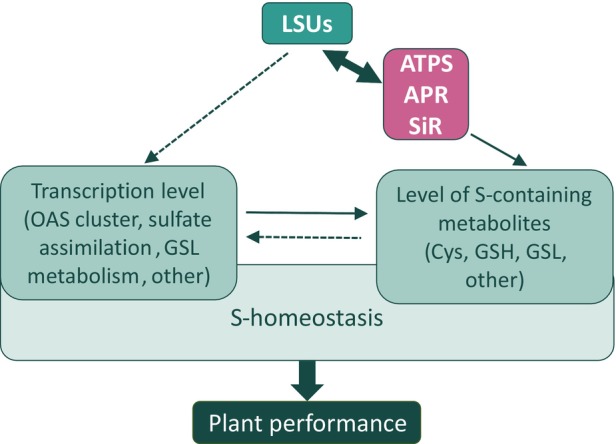
Model showing the role of LSU proteins in S metabolism homeostasis. Through binding to enzymes of the sulfate reduction pathway (ATP sulfurylase [ATPS], APS reductase [APR] and sulfite reductase [SiR]), LSU proteins positively affect their activity and influence the level of S‐containing metabolites. LSU proteins may also act as a signal of S‐status, triggering transcriptomic responses indirectly by altering S‐metabolite levels or directly through interactions with elements of the transcription machinery. The solid lines represent effects described in the literature and results, whereas the dashed lines represent hypothetical or indirect connections. Cys, cysteine; GSH, glutathione; GSL, glucosinolates; OAS, O‐acetylserine.

The interaction between all LSU proteins and a majority of the sulfate reduction pathway enzymes suggests that LSU proteins play a role in adjusting sulfate flux to sulfide depending on the demand and sulfate supply. All enzymes of the sulfate reduction pathway act in chloroplasts (Schwenn & Depka, 1977). Although the localization of LSU1 in chloroplasts has been proven (Garcia‐Molina et al., 2017), the mechanism by which LSU1 is imported into chloroplasts is yet unknown. The BiFC assay confirming the interaction between LSU1 and selected enzymes revealed localization near or inside chloroplasts in some instances, although most of the fluorescence was observed within the cytosol. The interaction between LSU1 and the chloroplastic enzyme FSD2 showed similar localization in the BiFC assay (Garcia‐Molina et al., 2017). However, in Arabidopsis, GFP‐LSU1 only partially co‐localized with chloroplasts, and this co‐localization occurred exclusively when the plants were grown under [dS] conditions (Garcia‐Molina et al., 2017). One can hypothesize that LSU proteins interact with chloroplastic proteins while still in the cytosol and then enter the chloroplasts as a complex. The unique feature of chloroplasts is their ability to transport folded proteins across a double membrane, made possible by the unusually large size of the protein translocon pore (Ganesan et al., 2018). Protein unfolding is not universally required for translocation and certain domains can retain their nonlinear structure during the process of entering chloroplasts. Currently, no data are available regarding the chloroplastic import of protein complexes. Interestingly, none of the LSU proteins interact with ATPS2, the isoform that has dual localization in plastids and the cytosol, due to alternative translational initiation (Bohrer et al., 2014). APS synthesized in the cytosol by ATPS2 is subsequently phosphorylated by APS kinase to form 3′‐phosphoadenosine 5′‐phosphosulfate (PAPS), which is used as a donor for sulfation reactions (Bohrer et al., 2014). ATPS2 is the only isoform that is not targeted by miR395 in the regulatory circuit and has no significant effect on cellular sulfate levels (Ai et al., 2016; Liang et al., 2010). In contrast, overexpression of the *ATPS1*, *ATPS3*, and *ATPS4* genes led to a reduction in sulfate levels, whereas their suppression resulted in sulfate accumulation in shoots (Ai et al., 2016). These three ATPS isoforms are, therefore, considered to be key regulators of the sulfate concentration in leaves. Interestingly, the expression of the two ATPS isoforms targeted by LSU proteins, ATPS1 and ATPS3, is driven by R2R3‐MYB transcription factors, which are known to activate both aliphatic and indolic GSL biosynthesis (Yatusevich et al., 2010). This finding indicated that the activation of sulfate by plastid ATPS is a bottleneck in the assimilative sulfate reduction pathway and the subsequent synthesis of secondary S‐metabolites. However, it has been reported that reduced function or loss‐of‐function alleles of *APR2* also lead to increased accumulation of total leaf sulfate (Chao et al., 2014). Quantitative trait locus (QTL) studies of sulfate accumulation in Arabidopsis revealed two key enzymes, ATPS1 and APR2 (Koprivova et al., 2013). The role of SiR in the reduction of inorganic sulfate to sulfide has long been considered insignificant for regulating flux in this pathway. Analysis of *sir* mutants revealed that optimal SiR activity is essential for normal growth and that its downregulation triggers severe adaptive reactions in both primary and secondary metabolism (Khan et al., 2010; Naumann et al., 2018). The accumulation of sulfate was again reported in the *sir1‐1* mutant. These data strongly suggest that impairment of any of the three steps in the sulfate reduction pathway leads to sulfate accumulation. This was also observed for the q‐lsu‐KO mutant, revealing the role of LSU proteins in the positive modulation of sulfate reduction pathway enzyme activities. Herein, we reveal the function of the LSU1 protein as a positive factor that directly binds to and enhances the enzymatic activity of the SiR enzyme. We cannot exclude the possibility that LSU proteins may also have other targets of S metabolism‐related enzymes, transporters, and regulators. Although we show here that all LSU proteins interact with the enzymes of the sulfate reduction pathway to the same extent, we can assume that they may have some specialization in target protein selection, as already demonstrated (Niemiro et al., 2020). The analysis of *LSU* tissue expression has primarily focused on LSU1 and LSU2, revealing distinct patterns of tissue‐specific expression that suggest specialized functions for these proteins. LSU1 is found broadly in root tissues but is highly concentrated in guard cells, implying a potential involvement in stomatal regulation. In contrast, LSU2 shows widespread expression in both leaves and roots, indicating a more general role across plant tissues (Garcia‐Molina et al., 2017). LSU1, LSU2, and LSU3 have been identified as immune‐related hub proteins, playing undefined roles in pathogen responses. These proteins possess relatively large and partially overlapping interactomes (Mukhtar et al., 2011; Wessling et al., 2014). Although LSU4 was not included in this study, earlier research has proposed its involvement in flower development (Myakushina et al., 2009). Interestingly, LSU proteins have been reported to interact with components of brassinosteroid signaling, jasmonate signaling, and the ethylene biosynthesis pathway suggesting their involvement in plant stress responses by influencing the signaling or synthesis of key phytohormones (Canales et al., 2023). We demonstrated here that LSU proteins play an important role in S homeostasis control and likely play a role in S status signaling as downstream transcriptomic responses are triggered. These responses may be induced either indirectly by generating changes in S‐metabolite levels sensed by, for example, SLIM1 or directly through interaction with transcription machinery elements. Among the known interaction partners of LSU proteins are transcription factors from various families (Arabidopsis Interactome Mapping Consortium, 2011; Frerigmann et al., 2014; Mugford et al., 2009). This suggests that LSU proteins may influence gene expression by modulating the activity of these transcription factors. Although the effects of *LSU* gene deletions are mainly observed under [nS] conditions, the overexpression of *LSU* genes improves tolerance to stresses like S deficiency or excess cadmium through manipulation of GSL metabolism (Li et al., 2023; Yang et al., 2023). Under heavy metal stress, LSU1 and LSU2 promote S assimilation through the induction of sulfate transporters at the transcriptional level, in addition to coordinating the flow of S from aliphatic GSL to S‐containing detoxification metabolites (Li et al., 2023). Interestingly, a recent genome‐wide association study in *Brassica juncea* identified a homolog gene of the Arabidopsis LSU2, as strongly associated with the gluconapin content, one of the main GSL (Tandayu et al., 2022). These findings imply that LSU proteins could play a role in regulating the biosynthesis of GSLs in *B. juncea*. Additionally, LSU3 has been identified as a protein partner of MYB51 transcription factor involved in the synthesis of indolic GSL (Frerigmann et al., 2014). The model of the proposed role of the LSU proteins in S metabolism is shown in Figure 9. In summary, although LSU proteins may not be essential or crucial for primary sulfate metabolism, they act as subtle regulators, fine‐tuning its efficiency depending on the plant's needs and influencing plant performance.

## EXPERIMENTAL PROCEDURES

### Plant material and growth conditions

All the plant lines used in this work were derivatives of the *A. thaliana* Col‐0 ecotype (denoted in this work as wild‐type, WT). The q‐lsu‐KO transgenic Arabidopsis line with a deletion in all four *LSU* genes was described previously (Piotrowska et al., 2024). The seedlings were cultivated *in vitro*. Seeds were dry sterilized as described previously (Zientara et al., 2009), plated onto half‐strength Hoagland's medium containing 0.8% agar, and stratified at 8°C for 2 days. The plates were placed vertically in a plant growth chamber with a photoperiod of 8 h of light and 16 h of darkness. Half‐strength Hoagland medium containing 1 mM sulfate was treated as a sulfur‐sufficient medium [nS]; in sulfur‐deficient medium [dS], MgSO_4_ was replaced with equimolar MgCl_2_. Seedlings were either grown for 10 days in [nS] media and transferred for 5 days into [nS] and [dS] media (transcriptomic studies) or germinated and grown in parallel in [nS] or [dS] media for 14 days (metabolomic studies).

### 3′RNA‐seq analysis

Total RNA was isolated separately from shoots and roots via TRI Reagent (Molecular Research Center, Cincinnati, OH, USA) according to the manufacturer's protocol (Chomczynski & Sacchi, 1987). Plant tissue was collected from at least 10 seedlings. The quality and concentration of RNA were evaluated via a NanoDrop ND‐100 (Thermo Fisher Scientific, Waltham, MA, USA) spectrophotometer. The Illumina libraries were prepared for 3′RNA‐seq via established protocol (Krzyszton et al., 2022), sequenced using NextSeq 500 and analyzed as described (Krzyszton et al., 2022). The differential expression (DE) of genes was analyzed using the DESeq2 (v1.8.2) package in R (v3.2.2) with the parameter alpha = 0.05. Genes with FDR‐adjusted *P* values <0.05 and FC values >1.5 were considered significantly different. Analysis of GO‐term enrichment was performed using the profiler R package (Kolberg et al., 2020) with the background set to genes detected in the experiment.

### Metabolite analysis

Shoots and roots were harvested and frozen in liquid nitrogen before the extraction of metabolites. For metabolite and ion determination, 50 mg aliquots of frozen, finely ground tissue were resuspended in 360 μL of ice‐cold methanol followed by 15 min of shaking at 950 rpm at 70°C. As a second extraction step, 200 μL of chloroform was added, and the samples were shaken at 950 rpm for 5 min at 37°C. Four hundred microliters of water were vortexed into the samples, which were subsequently centrifuged for 5 min at 20 800×*g*. The polar phase was transferred to a new tube, and the desired volumes were further aliquoted for LC–MS (300 μL) and ion chromatography (100 μL). The aliquots were dried overnight in a vacuum concentrator. LC–MS was performed as previously described (Perez de Souza et al., 2019, 2021). The concentrations of the metabolites were normalized by mass, and the data were subsequently filtered, autoscaled, and analyzed via the MetaboAnalyst 6.0 tool (https://www.metaboanalyst.ca/). For sulfate content determination, the evaporated polar phase was dissolved in high‐purity H_2_O and analyzed with a Dionex ICS‐3000 system as described previously (Apodiakou et al., 2024). OAS levels were determined after derivatization via an amino acid analysis protocol (Hubberten et al., 2012) and analyzed via the Dionex Summit HPLC system. Thiols were extracted via a previously described protocol (Hubberten et al., 2012). The labeled products were analyzed by HPLC using a LiChrospher 60 RP‐select B (5 μm) LiChroCART 125–4 chromatography column (Merck, Germany) in a Dionex Summit HPLC system.

### Sulfate uptake and flux analyses

Sulfate uptake and flux through the assimilation pathway were measured in seedlings grown in hydroculture for 14 days under sulfur‐sufficient (0.75 mM MgSO_4_) or sulfur‐limiting (0.015 mM MgSO_4_) conditions. Three microcuries of 0.2 mM [^35^S] ${\mathrm{SO}}_{4}^{2-}$ were added to the media, and the plants were incubated for 4 h in the light. Leaves and roots were separately extracted in a 10‐fold volume of 0.1 M HCl, and the ^35^S content was determined via scintillation counting to determine sulfate uptake and translocation to shoots. One hundred microliters of the extracts were used for determining the incorporation of different S‐containing compounds as described by Mugford et al. (2009).

### Gene cloning, vectors, and plasmid construction

The coding regions of *A. thaliana* genes were obtained from cDNA via the primers indicated in Table S4 and subsequently cloned Gateway™ pENTR/D‐TOPO to make an entry clone. Next, the LR reaction was carried out to construct an expression clone. All recombination reactions were carried out according to the manufacturer's protocols (Invitrogen, Thermo Fisher Scientific). The list of constructs and vectors used is available in Table S5.

### Protein–protein interaction assays

Yeast two‐hybrid (Y2H) experiments were performed with the indicated ORFs cloned into pDEST‐GAD or pDEST‐GBK as described previously (Niemiro et al., 2020). Bimolecular fluorescence complementation (BiFC) assays were performed to determine the protein interactions *in planta*. Briefly, *N. benthamiana* leaves were agroinfiltrated with *Agrobacterium tumefaciens* (strain GV3101) and transformed with combinations of the respective plasmids. The plasmids encoded either the N‐terminal (pSITE‐nEYFP‐C1) section of YFP linked to LSU1 or the C‐terminal section of YFP (pSITE‐cEYFP‐N1) linked to the C‐terminal part of ATPS1, APR1, or SiR. *A. tumefaciens* bacteria were grown overnight at 28°C in yeast extract broth (YEB) supplemented with 100 μg mL^−1^ rifampicin and 50 μg mL^−1^ spectinomycin (BioShop, Canada) prior to agroinfiltration. Microscopy observations were made via a Nikon Eclipse TE2000‐E inverted confocal microscope (Nikon Corporation, Japan). Interactions were tested using a 488 nm laser (Sapphire 488–20 CDRH; Coherent Inc., USA) and a 515/30 filter. For chloroplast visualization, a 615/75 filter was used. The image data were analyzed via an EZ‐C1 3.90 FreeViewer (Nikon Corporation, Tokio, Japan).

### Protein overexpression and purification

*Escherichia coli* Rosetta (DE3) cells were transformed with N‐terminally His‐tagged SiR, LSU1 or ZIP expression constructs (see Table S5) and grown to log phase (OD600 0.4–0.8) at 30°C in 3 mL Luria–Bertani (LB) media supplemented with ampicillin (100 μg mL^−1^) and chloramphenicol (50 μg mL^−1^). Protein expression induction was initiated with 1 mM isopropyl β‐d‐1‐thiogalactopyranoside (IPTG) and allowed to proceed for 1 h (for SiR) or 3 h (for LSU1 and ZIP). Pelleted cells were resuspended in extraction buffer [20 mm sodium‐phosphate buffer (pH 7.4), 0.3 M NaCl, 10 mM imidazole, 1 mm phenylmethylsulfonyl fluoride (PMSF), 1 mm ethylenediaminetetraacetic acid (EDTA), and 1× Complete Protease Inhibitor Cocktail Tablet (Roche)], sonicated, and the resulting lysates were rotated with HIS‐Select HF Nickel Affinity (Sigma–Aldrich) o/n at 4°C. The matrix was washed five times with wash buffer [20 mM sodium phosphate buffer (pH 7.4), 0.3 M NaCl, and 10 mM imidazole] and eluted with elution buffer [20 mM sodium phosphate buffer (pH 7.4), 0.3 M NaCl, and 250 mM imidazole].

### SiR enzymatic assay

SiR activity was measured via a previously described protocol (Naumann et al., 2018). Enzymatic kinetic studies of the recombinant His‐SiR confirmed the concentrations used in the original assay (Figure S9). Approximately 7–10 μg of purified His‐tagged SiR was added to 150 μL of the master mix (5% BSA, 25 mM HEPES, pH 7.5, 1 mM Na_2_SO_3_, 5 mM O‐acetylserine, ~10 μg of O‐acetylserine(thiol)lyase enzyme, 10 mM DTT, 30 mM NaHCO_3_, 15 mM Na_2_S_2_O_4_, and 5 mM methyl viologen) for 1 h at RT in the dark. The reaction was stopped by adding 350 μL of Gaitonde reagent (250 mg of ninhydrin dissolved in 16 mL of acetic acid and 4 mL of 12 N HCl) (Gaitonde, 1967). The cysteine produced in the supernatant was detected by a ninhydrin reaction at 100 °C for 10 min. The mixture was subsequently allowed to cool at RT for 10 min, and the optical density at 560 nm was recorded. To allow interaction, purified recombinant His–LSU1 (100 μg) or His–ZIP (100 μg) was incubated with the His‐tagged SiR protein in 50 μL of PBS at room temperature for 30 min prior to enzymatic activity determination.

### Statistical test

The statistical tests performed are indicated in the figure legends or the descriptions of the methods.

### ACCESSION NUMBERS

The *A. thaliana* genes included in this study are as follows: AT3G49580 (*LSU1*), AT5G24660 (*LSU2*), AT3G49570 (*LSU3*), AT5G24655 (*LSU4*), AT3G22890 (*ATPS1*), AT1G19920 (*ATPS2*), AT4G14680 (*ATPS3*), AT5G43780 (*ATPS4*), AT4G04610 (*APR1*), AT1G62180 (*APR2*), AT4G21990 (*APR3*), AT5G04590 (*SiR*), AT5G48850 (*SDI1*), AT2G44460 (*BGLU28*), AT5G26220 (*GGCT2;1*), AT5G10180 (SULTR2;1), AT3G12520 (SULTR4;2), AT1G18590 (SOT17), AT4G12030 (BAT5), AT1G16410 (*CYP79F1*), AT1G16400 (CYP79F2), AT5G23010 (*MAM1*), AT3G19710 (*BCAT4*), AT5G51100 (*FSD2*), and AT3G44320 (*NIT3*).

## AUTHOR CONTRIBUTIONS

JP and AW designed the research and performed the experiments; MO provided suggestions on the experiments and discussed the data; MK assisted with the RNAseq preparation and data analysis; AA helped with the measurements of S‐containing metabolites; SA performed the LC–MS metabolite analysis; JMLR performed the S flux experiment; JP, AS, and AW wrote and edited the manuscript; and SK and RH provided suggestions and assisted with the manuscript. The manuscript has been read, revised, and approved by all the listed authors.

## CONFLICT OF INTEREST STATEMENT

The authors declare no conflicts of interest.

## Supporting information


**Figure S1.** Comparative analysis of transcriptome profiles of WT and q‐lsu‐KO mutant shoots and roots under normal conditions [nS] and 5 day S deficiency [dS]. (a, b) Volcano plots showing comparison of transcript level in [nS] versus [dS] conditions or in q‐lsu‐KO mutant versus WT in shoots (a) and roots (b). The fold change (log2) is plotted on the x‐axis, and the adjusted *P*‐value (negative log10) is plotted on the y‐axis. Light gray dots represent all genes while the colored dots represent significantly changed genes (fold change ≥1.5 and FDR ≤0.05): in red—increased level, while in blue—decreased level.
**Figure S2.** Comparative analysis of metabolome profiles of WT and q‐lsu‐KO mutant shoots and roots of plants grown for 14 days on either S‐sufficient [nS] or S‐deficient [dS] conditions. Volcano plots showing comparison of metabolite level in q‐lsu‐KO mutant versus WT in [nS] and [dS] in shoots (a) and roots (b). The fold change (log2) is plotted on the x‐axis, and the adjusted *P*‐value (negative log10) is plotted on the y‐axis. Light gray dots represent all metabolites while the colored dots represent significantly changed metabolites (fold change ≥1.5 and FDR ≤0.05): in red—increased level, while in blue—decreased level.
**Figure S3.** Negative controls for Bimolecular Fluorescence Complementation (BiFC) assay. Coexpression of the YFP section with LSU1, APS1, APR1, and SiR fusions with appropriate YFP section showing no green spots. cY and nY represent the C‐terminal and N‐terminal sections of YFP, respectively. Scale bars: 10 μm.
**Figure S4.** The gel showing the production and purification of recombinant His‐SIR and His‐LSU1 from *Escherichia coli*. The expected sizes of the proteins—SiR: 68 kDa and LSU1: 11 kDa.
**Figure S5.** Y2H screen of the interaction between SiR and LSU1 and bZIP domain of ABF3. Below is the gel showing the production and purification of recombinant His‐bZIP from *Escherichia coli*. The expected size of the protein is 11 kDa.
**Figure S6.** Recombinant His‐SiR protein purified from *Escherichia coli* is enzymatically active. SiR activity of increasing concentrations of recombinant His‐SiR was spectrophotometrically determined and represented. Error bars correspond to the SD of three technical repetitions. The experiment was repeated three times with similar results.
**Figure S7.** SiR in vitro activity in time in the presence of either LSU1 or ZIP proteins. Error bars correspond to the SD of three technical repetitions. The experiment was repeated three times with similar results.
**Figure S8.** Schematic summary of S‐related gene and metabolite changes in WT by S‐deficiency [dS] compared to changes in q‐lsu‐KO mutant in normal S conditions [nS]. Only statistically significant changes are shown; in red increased level; in blue decreased level; in white no change; dashed blue boxes show decreased level though not statistically significant; Cys‐Gly, cysteinylglycine; GSH, glutathione; γ‐EC, gamma‐glutamylcysteine.
**Figure S9.** Enzyme kinetics of the recombinant His‐SiR enzyme purified from *Escherichia coli*. Specific activities of the enzyme at various concentrations of: electron donor methyl‐viologen (a), substrate sulfite (b), and in different temperatures of reaction (c). Michaelis–Menten plots are shown. All measurements were made in duplicate in three independent experiments and represented as means with standard deviation.

**Table S1.** The list of differentially expressed genes (DEGs) between genotypes and between conditions.

**Table S2.** The detailed list of enriched GO categories identified in the compared datasets from RNAseq.

**Table S3.** The processed metabolic data with FDR and log2 fold‐change values.

**Table S4.** The list of primers used in this work.

**Table S5.** The list of vectors and plasmids used in this work.

## Data Availability

The datasets generated and analyzed during the current study are available from NCBI's Gene Expression Omnibus (Edgar et al., 2002) and are accessible through GEO series accession number GSE 271678. Other data that support the findings of this study are available from the corresponding author upon request.
