# Supplementary material for: Analysis of the quadruple lsu mutant reveals molecular determinants of the role of LSU proteins in sulfur assimilation in Arabidopsis
Source: Plant J. 2024 Nov 29;120(6):2919–36. doi: 10.1111/tpj.17155 (PMC11658185; doi:10.1111/tpj.17155)
Supplement: Supplementary file 1 — Figure S1. Comparative analysis of transcriptome profiles of WT and q‐lsu‐KO mutant shoots and roots under normal conditions [nS] and 5 day S deficiency [dS]. (a, b) Volcano plots showing comparison of transcript level in [nS] versus [dS] conditions or in q‐lsu‐KO mutant versus WT in shoots (a) and roots (b). The fold change (log2) is plotted on the x‐axis, and the adjusted P‐value (negative log10) is plotted on the y‐axis. Light gray dots represent all genes while the colored dots represent significantly changed genes (fold change ≥1.5 and FDR ≤0.05): in red—increased level, while in blue—decreased level. Figure S2. Comparative analysis of metabolome profiles of WT and q‐lsu‐KO mutant shoots and roots of plants grown for 14 days on either S‐sufficient [nS] or S‐deficient [dS] conditions. Volcano plots showing comparison of metabolite level in q‐lsu‐KO mutant versus WT in [nS] and [dS] in shoots (a) and roots (b). The fold change (log2) is plotted on the x‐axis, and the adjusted P‐value (negative log10) is plotted on the y‐axis. Light gray dots represent all metabolites while the colored dots represent significantly changed metabolites (fold change ≥1.5 and FDR ≤0.05): in red—increased level, while in blue—decreased level. Figure S3. Negative controls for Bimolecular Fluorescence Complementation (BiFC) assay. Coexpression of the YFP section with LSU1, APS1, APR1, and SiR fusions with appropriate YFP section showing no green spots. cY and nY represent the C‐terminal and N‐terminal sections of YFP, respectively. Scale bars: 10 μm. Figure S4. The gel showing the production and purification of recombinant His‐SIR and His‐LSU1 from Escherichia coli. The expected sizes of the proteins—SiR: 68 kDa and LSU1: 11 kDa. Figure S5. Y2H screen of the interaction between SiR and LSU1 and bZIP domain of ABF3. Below is the gel showing the production and purification of recombinant His‐bZIP from Escherichia coli. The expected size of the protein is 11 kDa. Figure S6. Recombinant His‐ [file TPJ-120-2919-s005.pdf]

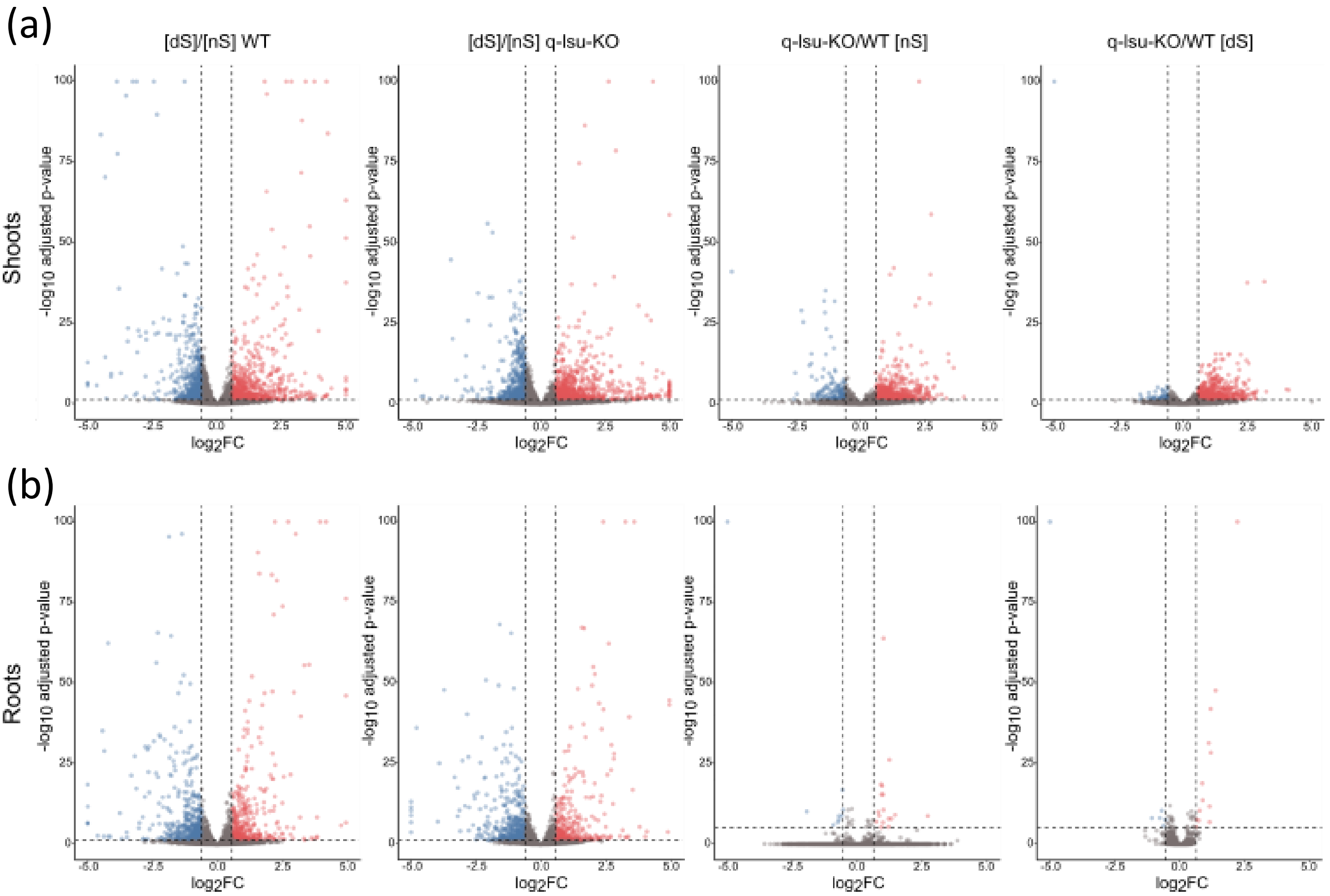

Figure S1. Comparative analysis of transcriptome profiles of WT and q-lsu-KO mutant shoots and roots under normal conditions [nS] and 5-day S deficiency [dS].

(a,b) Volcano plots showing comparison of transcript level in [nS] vs [dS] conditions or in q-lsu-KO mutant vs WT in shoots (a) and roots (b). The fold change (log2) is plotted on the x-axis, and the adjusted p-value (negative log10) is plotted on the y-axis. Light gray dots represent all genes while the colored dots represent significantly changed genes (fold change ≥ 1.5 and FDR ≤ 0.05): in red – increased level while in blue – decreased level.

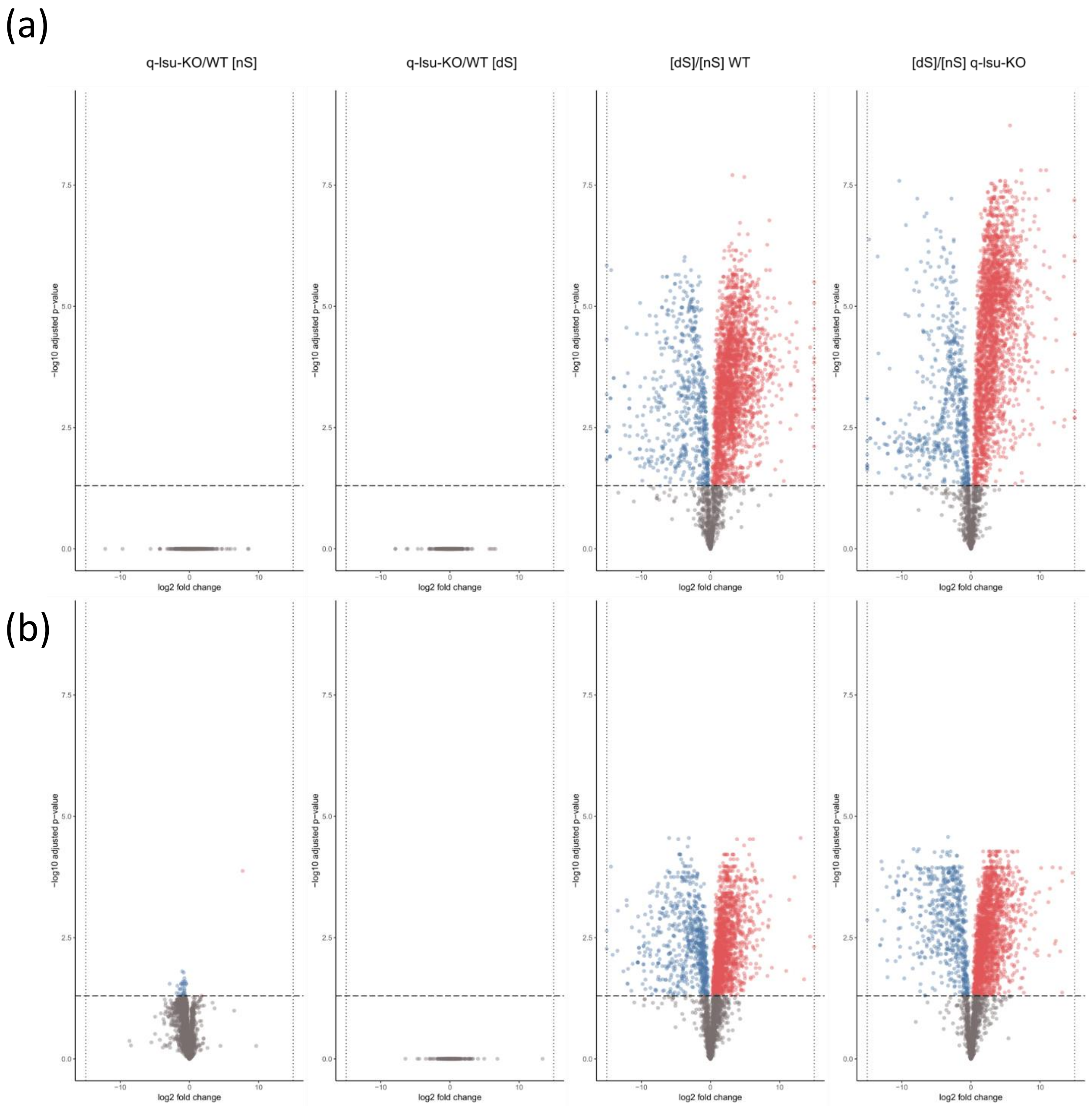

Figure S2. Comparative analysis of metabolome profiles of WT and q-lsu-KO mutant shoots and roots of plants grown for 14 days on either S-sufficient [nS] or S-deficient [dS] conditions.
Volcano plots showing comparison of metabolite level in q-lsu-KO mutant vs WT in [nS] and [dS] in shoots (**a**) and roots (**b**). The fold change (log2) is plotted on the x-axis, and the adjusted p-value (negative log10) is plotted on the y-axis. Light gray dots represent all metabolites while the colored dots represent significantly changed metabolites (fold change ≥ 1.5 and FDR ≤ 0.05): in red – increased level while in blue – decreased level.

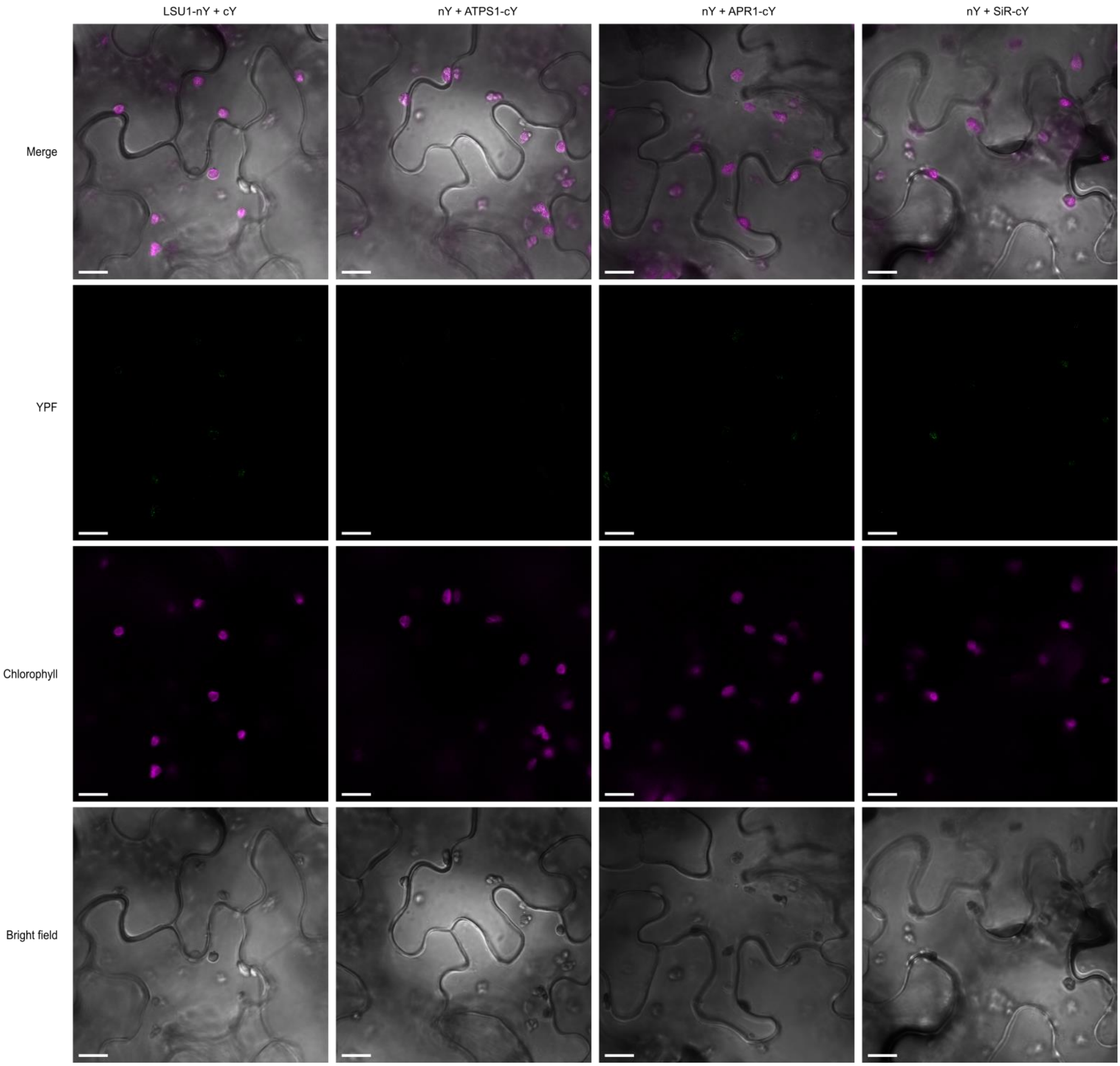

Figure S3. Negative controls for Bimolecular Fluorescence Complementation (BiFC) assay. Coexpression of YFP section with LSU1, APS1, APR1, and SiR fusions with appriopriate YFP section show no green spots. cY and nY represent the C-terminal and N-terminal sections of YFP, respectively. Scale bars: 10 µm.

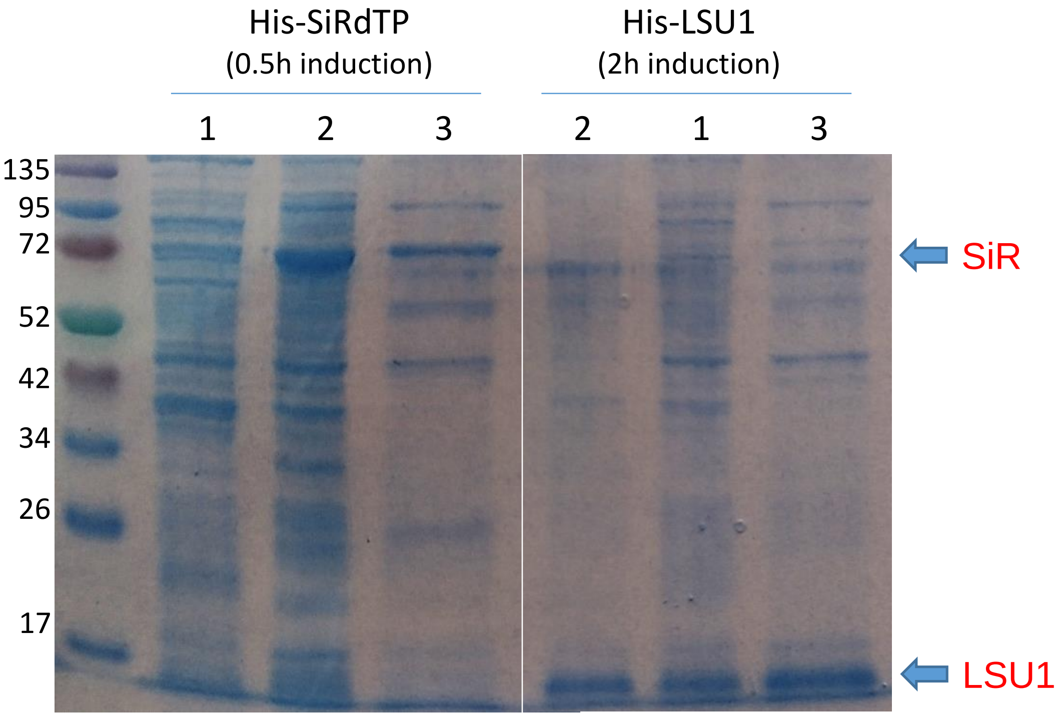

1 – supernatant after sonication
2 – pelet after sonication
3 – elution

Figure S4. The gel showing the production and purification of recombinant His-SIR and His-LSU1 from *Escherichia coli*. The expected sizes of the proteins – SiR: 68 kDa and LSU1: 11 kDa.

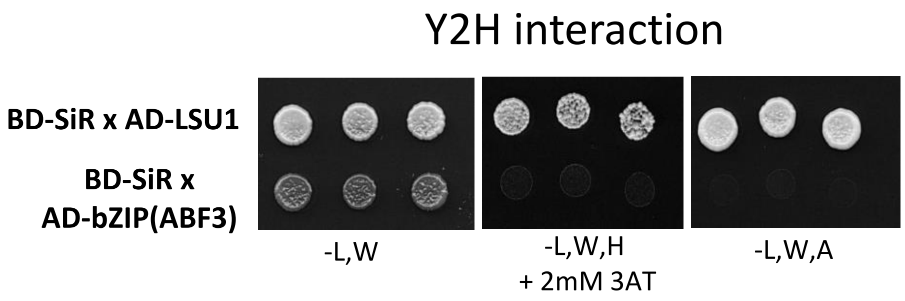

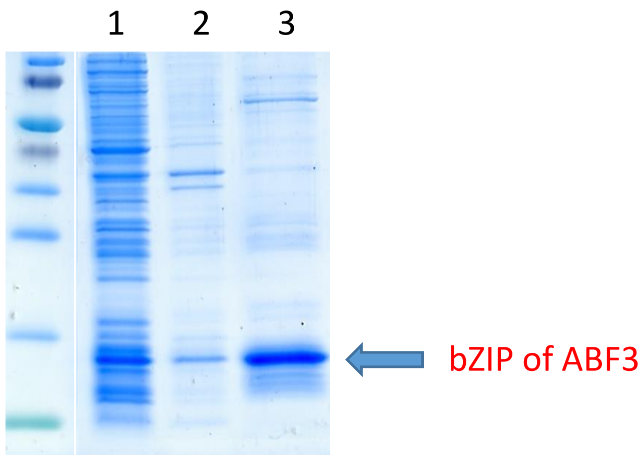

1 – supernatant after sonication
2 – pelet after sonication
3 – elution

Figure S5. Y2H screen of the interaction between SiR and LSU1 and bZIP domain of ABF3. Below the gel showing the production and purification of recombinant His-bZIP from *Escherichia coli*. The expected size of the protein is 11 kDa.

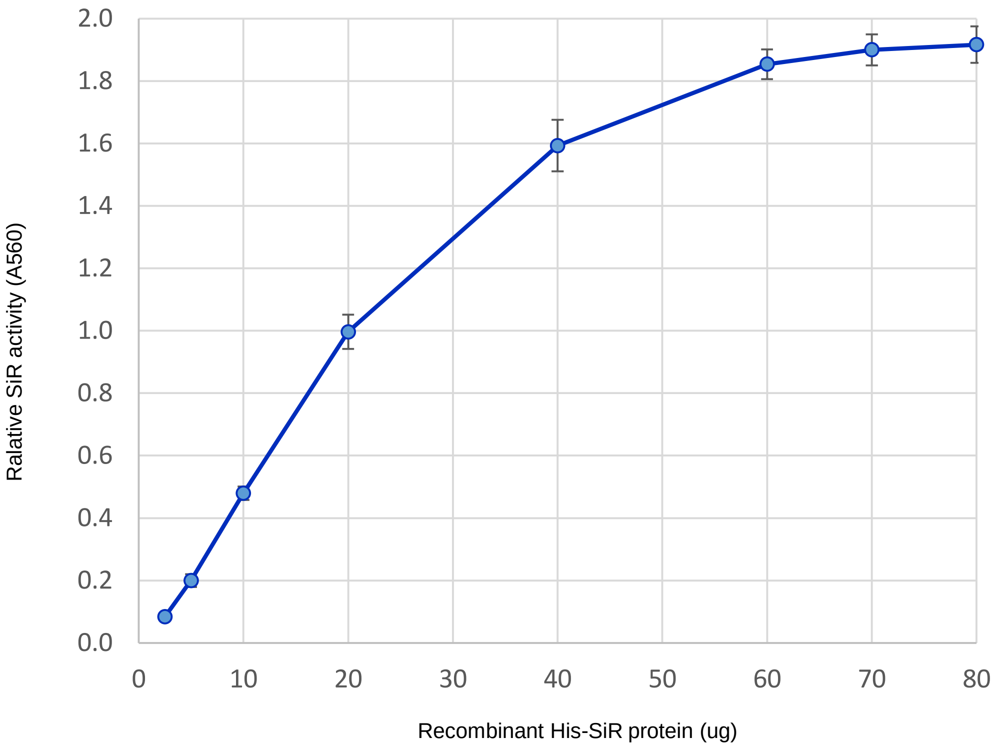

Figure S6. Recombinant His-SiR protein purified from *E. coli* is enzymatically active. SiR activity of increasing concentrations of recombinant His-SiR was spectrophotometrically determined and represented. Error bars correspond to the SD of three technical repetitions. The experiment was repeated three times with similar results.

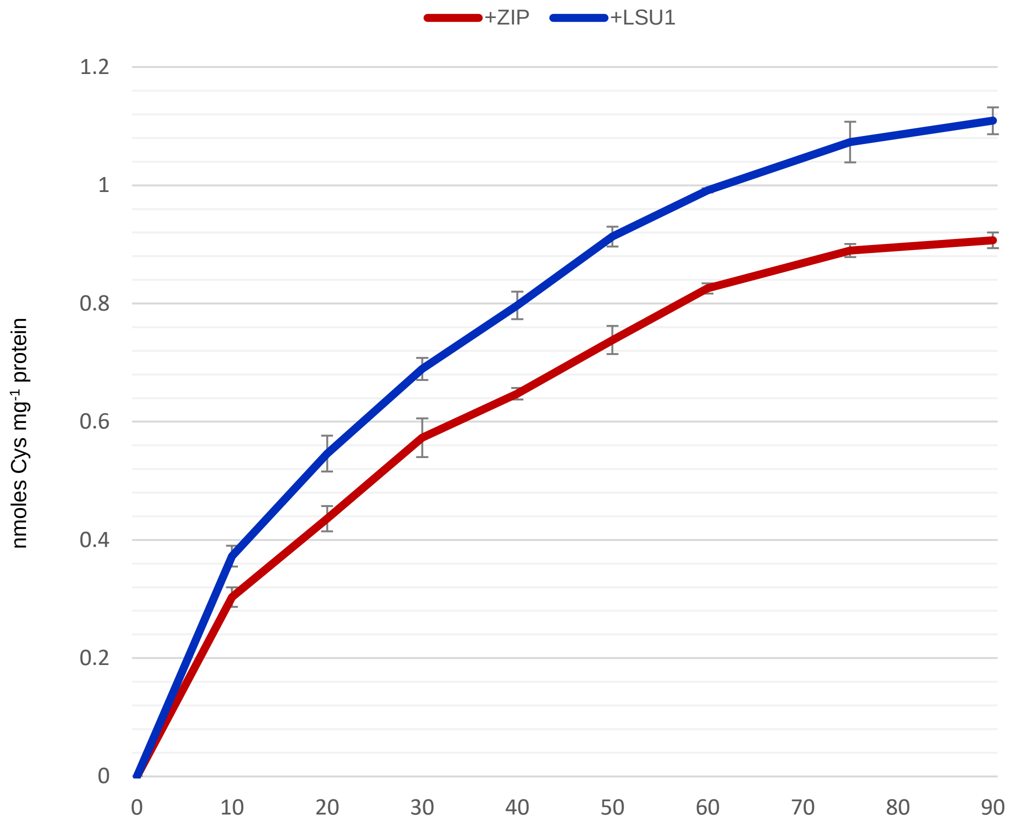

Figure S7. SiR in vitro activity in time in the presence of either LSU1 or ZIP proteins. Error bars correspond to the SD of three technical repetitions. The experiment was repeated three times with similar results.

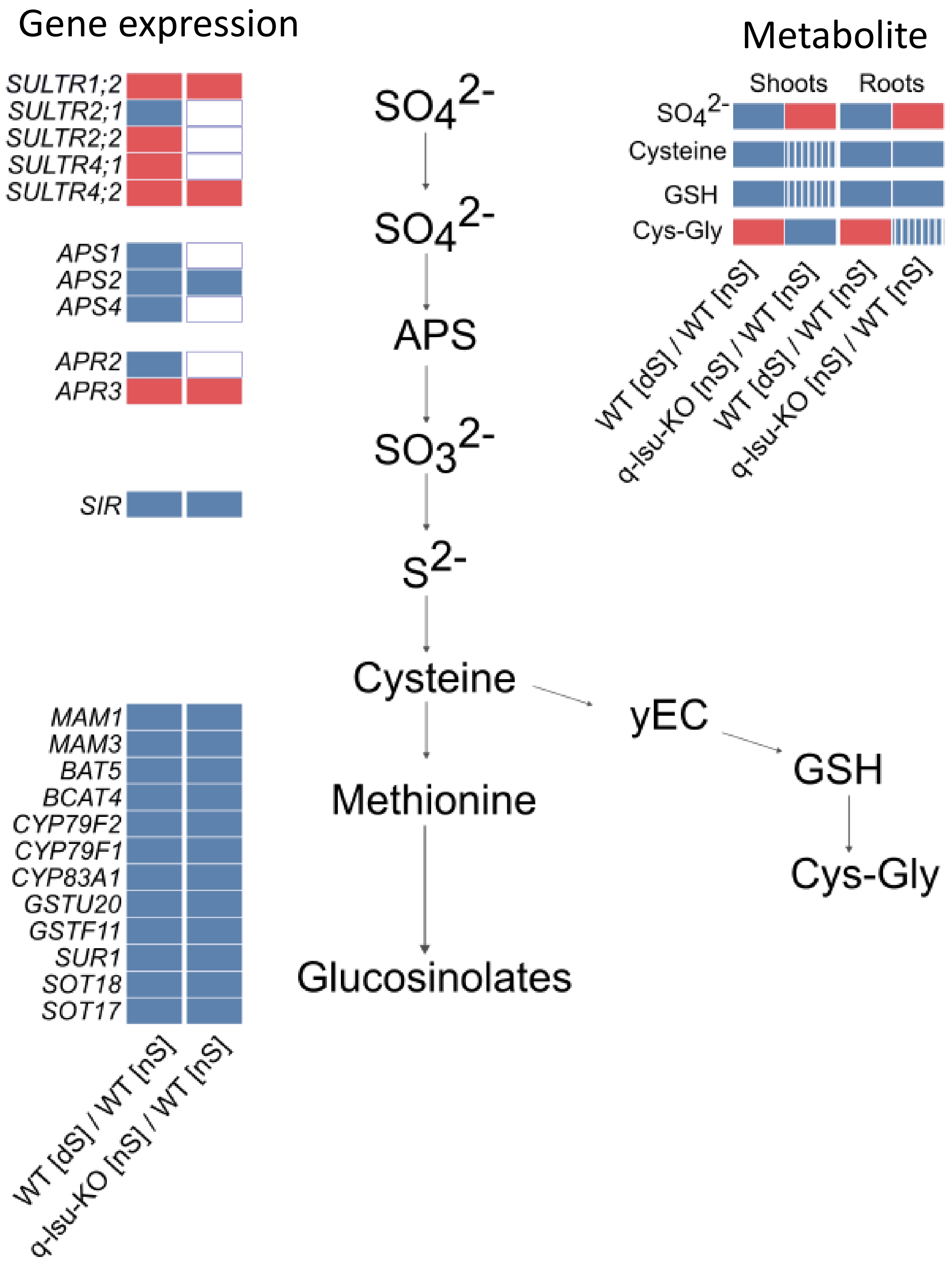

Figure S8. Schematic summary of S-related gene and metabolite changes in WT by S-deficiency [dS] compared to changes in q-lsu-KO mutant in normal S conditions [nS]. Only statisticaly significant changes are shown; in red increased level; in blue decreased level; in white no change; dashed blue boxes show decreased level though not statisticaly significant; GSH – glutathione; γ-EC - gamma-glutamylcysteine; Cys-Gly - cysteinylglycine

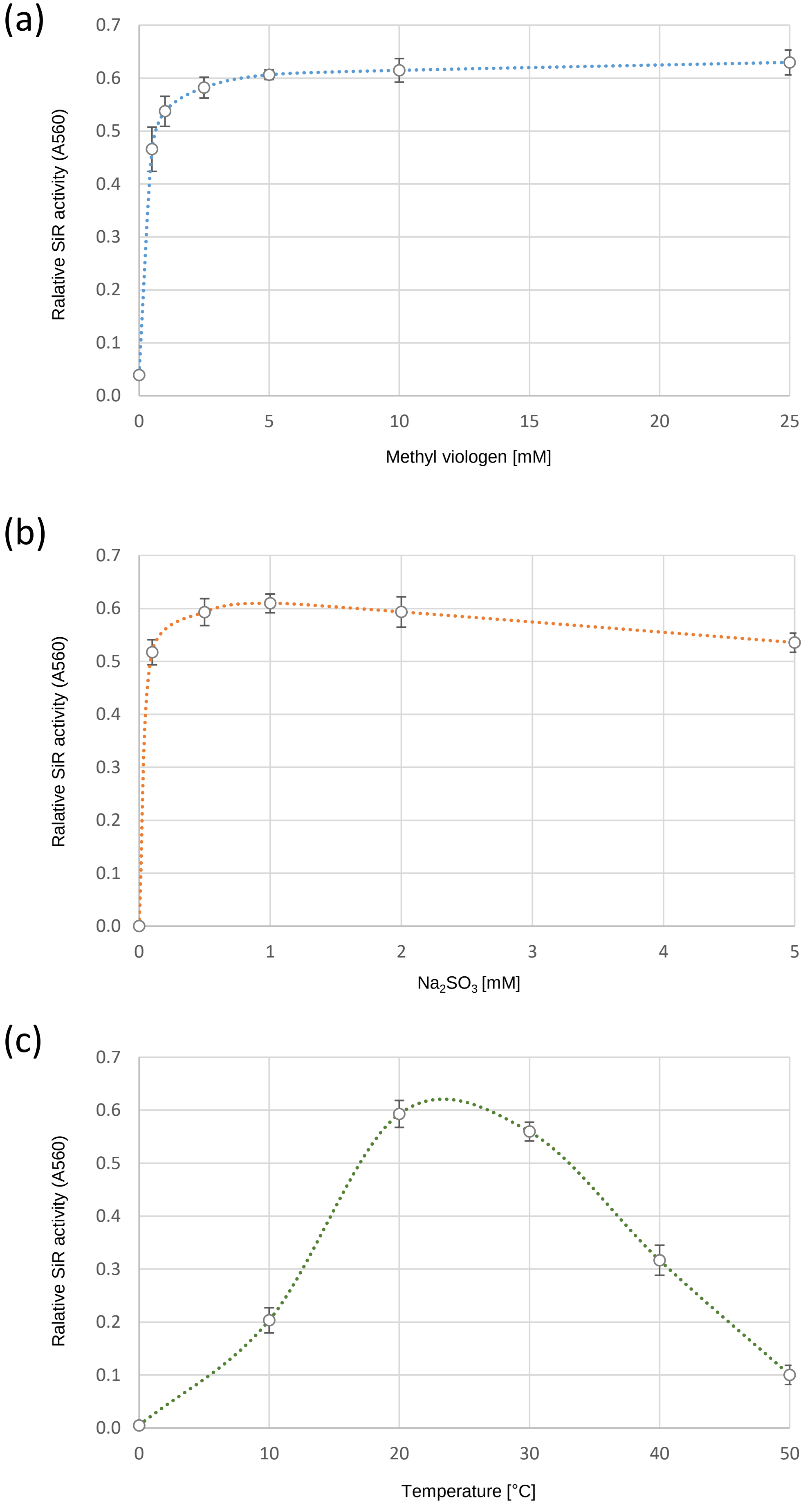

Figure S9. Enzyme kinetics of the recombinant His-SiR enzyme purified from *E. coli*. Specific activities of the enzyme at various concentrations of: electron donor methyl-viologen (a), substrate sulfite (b), and in different temperatures of reaction (c). Michaelis–Menten plots are shown. All measurements were made in duplicate in three independent experiments and represented as means with standard deviation.
